# Effects of an intervention combining warm therapy with a digital distraction app on pain, stress, and satisfaction during intravenous catheterization in South Korea: a randomized controlled trial

**DOI:** 10.7586/jkbns.25.020

**Published:** 2025-05-14

**Authors:** Jae-Kyeum Lee, Ki-Yong Kim, Yean-Hee Jeong, Yu-Jin Lee, Min-Ho Lee, Myung-Haeng Hur

**Affiliations:** 1 College of Nursing, Gimcheon University, Gimcheon, Korea; 2 College of Nursing, Eulji University, Ujeongbu, Korea; 3 Department of Culture and Arts Management, Hannam University, Daejeon, Korea

**Keywords:** Catheterization, Pain, Patient satisfaction, Job satisfaction, Augmented reality

## Abstract

**Purpose:**

This study aimed to evaluate the effects of an intervention combining warm therapy (via a thermoelectric-element tourniquet) and a distraction-based approach (via an augmented reality-based app known as TWINKLE) on pain, stress, and satisfaction during intravenous catheterization in adults.

**Methods:**

A randomized controlled trial was conducted in South Korea with 93 healthy adults who were randomly assigned to one of three groups: the experimental group (TWINKLE app with warm therapy), the comparison group (warm therapy only), and the control group (no treatment). Participants’ pain, stress, and satisfaction, as well as practitioner satisfaction, were measured after the intervention.

**Results:**

Pain scores differed significantly among the three groups (F = 5.68, *p* = .005), with the experimental group showing significantly lower scores than the control group (*p* = .003). Stress levels were also significantly lower in the experimental group than in the other groups (F = 9.42, *p* < .001). Participant satisfaction was highest in the experimental group (F = 17.65, *p* < .001), while nurse satisfaction was significantly higher in the comparison group than in the experimental and control groups (F = 67.91, *p* < .001), suggesting that the additional distraction intervention may have increased nurses’ workload.

**Conclusion:**

Combining digital distraction with warm therapy using a thermoelectric-element tourniquet effectively reduces pain and stress while improving patient satisfaction during intravenous catheterization. Further research is needed to optimize this approach, with a particular focus on targeting digital distraction interventions to patients with higher levels of procedural anxiety and finding ways to minimize practitioner workload.

## INTRODUCTION

Intravenous (IV) catheterization is a routine clinical procedure commonly performed during hospitalization for purposes such as administering medications, fluids, or contrast media. Compared to intramuscular or subcutaneous injections, venipuncture is more efficient and allows for faster drug effects. Its versatility in enabling fluid therapy, medication delivery, and diagnostic testing makes it an essential component of inpatient care [1,2]. Nevertheless, repeated catheterizations are a common source of physical and psychological discomfort for patients [3]. Because IV catheterization is not only a physical procedure but also directly triggers an emotional response from patients, it can cause anxiety and fear in addition to physical pain [4]. Some patients have reported particularly strong fears of venipuncture because of previous experiences of IV infusion [5]. IV catheterization also causes significant stress for healthcare workers and nurses due to the difficulty in securing venous access. When catheterization is difficult, repeated venipunctures may be required, which further exacerbates distress for patients and work burden among healthcare workers [6]. As such, some authors [7,8] have highlighted the need for nursing interventions to reduce pain and stress during IV catheterization and enable easier and more effective venous access.

Various interventions have been devised to reduce the pain and stress caused by IV catheterization. These include pharmacological approaches, such as lidocaine patches [9], lidocaine ointment [10], and aroma hand massage [11], which have shown significant pain-relieving effects. However, caution is required when using local anesthetic-based methods due to potential adverse effects, such as skin irritation, rash, discomfort, and delayed onset of action [10]. In addition, a range of non-pharmacological interventions has been applied to alleviate patients’ anxiety and pain during venipuncture. Among non-pharmacological approaches, distraction techniques—such as music interventions [12], video games [13], and virtual reality (VR) [14]—have been widely used to reduce patients’ anxiety and pain.

Distraction-based interventions focus attention on visual or auditory stimuli to reduce the perception of the pain. It is also a method to reduce psychological problems such as pain and anxiety. Distraction-based interventions are non-pharmacological method essential for reducing pain, incompetence, and fear that occur during hospital treatment [15]. Non-medicinal interventions used to alleviate pain from IV cannulation include counter-stimulation techniques such as warm therapy and cryotherapy [16]. Warm therapy seeks to provide a soothing effect by blocking pain transmission based on the gate control theory; it helps to alleviate ischemia-related pain by inducing vasodilation. Cryotherapy reduces pain by inducing vasoconstriction, restricting local cellular metabolism [17]. However, warm therapy- and cryotherapy-based interventions have suffered from issues, such as difficulty maintaining a constant temperature [18], slowness in achieving appropriate temperatures for pain reduction [19], and inapplicability in clinical settings due to the need for prior preparation (before IV cannulation can occur) [8]. Combining the two interventions can address limitations such as distraction-based interventions may be effective only for children and specific patient groups, and the difficulty of maintaining a constant temperature in warm therapy [7,8].

Recently, the thermoelectric-element tourniquet (TEET) has been developed and applied as a novel non-pharmacological intervention to overcome these limitations [8]. TEET is a device that combines the function of a previously developed thermoelectric element (TEE) wristband with a tourniquet function and a thermostatic device. It is able to maintain a constant target temperature, making it more efficient than regular warm therapy; it has been shown to reduce pain effectively during IV catheterization [8]. With the growing emphasis on the need for nursing interventions to relieve pain associated with IV catheterization, the TEET has recently been developed and applied in clinical settings. TEET maintains a constant target temperature, offering a practical advantage over conventional warm or cold packs by enabling the application of thermotherapy and cryotherapy in a more efficient manner. In a previous study, heat therapy using a TEE band resulted in significantly higher patient satisfaction in the intervention group compared to the control group, indicating the potential utility of thermoelectric-based interventions [16]. In a subsequent randomized controlled trial using the improved, flexible modified TEET, cryotherapy was shown to significantly reduce pain, and thermotherapy increased regional venous blood flow, leading to improved satisfaction among both patients and practitioners [8].

However, previous TEET-related interventions have primarily focused on alleviating physical pain, while psychological anxiety and stress during IV catheterization also represent critical aspects that warrant nursing intervention [8]. To address this, the present study combined thermotherapy using TEET with a digital distraction intervention, employing the TWINKLE app, which is designed to divert patients’ attention and reduce negative perceptions of the IV catheterization process.

This study aimed to evaluate the effects of a combined intervention using thermoelectric-based thermotherapy and a digital distraction strategy on pain, stress, patient satisfaction, and practitioner satisfaction among healthy adults undergoing IV catheterization. Healthy adults were selected as study participants to minimize potential confounding variables related to underlying medical conditions or medication use, allowing for a clearer assessment of the pure effects of the intervention. Pain and stress were chosen as outcome variables to capture immediate physiological and psychological responses to IV catheterization, while patient satisfaction was included as an indicator of perceived nursing care quality. Additionally, practitioner satisfaction was assessed to evaluate the clinical feasibility and applicability of the intervention in real-world settings. Unlike previous studies that examined each intervention in isolation, this study is significant in that it assesses the effects of a multidimensional nursing intervention that simultaneously addresses both physical and psychological aspects of patient care. This integrated approach is expected to provide new insights into nursing interventions that simultaneously address physical and psychological responses during IV catheterization.

## METHODS

### 1. Study Design

**Figure 1.**
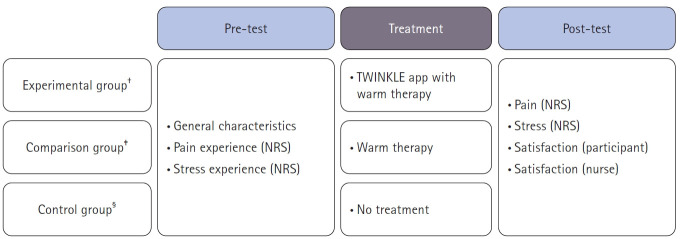
Overview of the study design and intervention process. app = Application; NRS = Numerical rating scale. ^†^Experimental group = TWINKLE app with warm therapy; ^‡^Comparison group = Warm therapy only; ^§^Control group = No intervention.

This was a randomized controlled trial for the purpose of investigating differences in the participant’s pain, stress, and satisfaction as well as the practitioner’s satisfaction when applying TEET and TWINKLE during IV catheterization in healthy adults (Figure 1).

### 2. Participants

The participants in this study were healthy adults residing in South Korea who voluntarily responded to a recruitment notice posted on campus bulletin boards at Gimcheon University in Gimcheon, South Korea. All participants agreed to participate after receiving a detailed explanation of the study’s purpose and procedures. The inclusion criteria were as follows: adults aged 19 or older who were capable of effective communication, had no visible or reported issues at the venipuncture site, and had experienced IV catheterization within the past six months. The exclusion criteria were: individuals with impaired consciousness or disorientation; those currently undergoing treatment for physical or mental illnesses; individuals taking medications that could influence pain or stress perception, such as anxiolytics, sedatives, cold medicine, or analgesics; and individuals with known sensitivity to temperature changes. Additionally, participants who had prior exposure to either the TEET or the TWINKLE app were excluded to ensure unbiased evaluation of the intervention’s effects.

**Figure 2.**
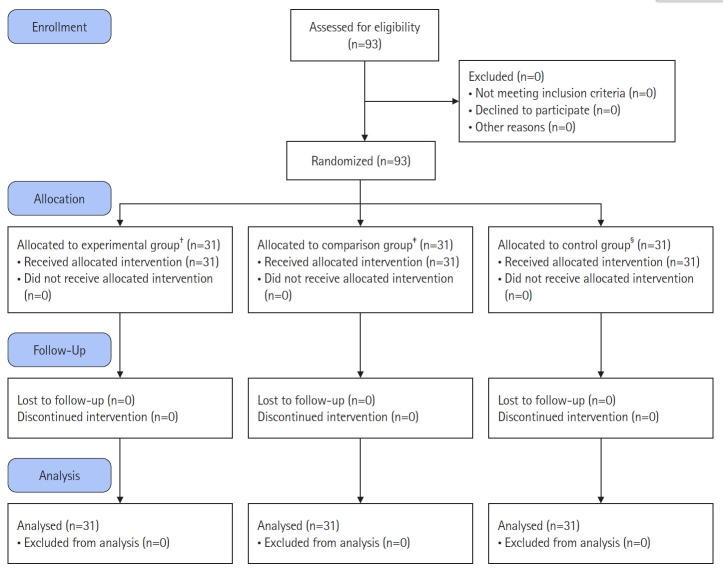
Study Flow diagram. ^†^Experimental group =TWINKLE application with warm therapy; ^‡^Comparison group = Warm therapy only; ^§^Control group = No intervention.

We calculated the required sample size using G*Power 3.1.9 based on an alpha level of .05, a statistical power (1–β) of .80, and an effect size of .35 (F-test) based on previous studies [8]. With three groups to compare, the minimum required sample size was 84 participants. To account for a potential 10% dropout rate, we recruited 93 participants. After recruitment, participants were randomly assigned to one of the three study groups (experimental, comparison, or control) using a random number generator in Microsoft Excel 2019, with 31 participants allocated to each group (Figure 2).

### 3. Procedures

#### 1) Experimental group

Participants in the experimental group received warm therapy using the TEET, followed by venipuncture while using the TWINKLE app, a digital distraction tool based on augmented reality. The intervention was conducted in a laboratory setting at a controlled ambient temperature (22°C~25°C), with the participant in a seated position. The TEET was applied 10~12 cm above the selected venipuncture site, maintaining a temperature of 40°C~45°C for 10~60 seconds. While warm therapy was applied, the TWINKLE app was used to provide visual distraction. To ensure methodological consistency with previous studies on thermoelectric interventions [8], IV catheterization was performed using an 18-gauge (18G) angiocatheter. This catheter size was also chosen to elicit a more noticeable physical stimulus, allowing clearer assessment of pain and satisfaction. A nurse with at least three years of clinical experience performed all procedures. After catheterization, participants completed questionnaires measuring perceived pain, stress, and satisfaction.

#### 2) Comparison group

Participants in the comparison group received warm therapy using the TEET under the same environmental conditions as the experimental group. The TEET was applied for 10~60 seconds at a temperature of 40°C~45°C before venipuncture, but without the use of the TWINKLE app. After warm therapy, IV catheterization was performed using the same procedure and equipment as in the experimental group. Participants subsequently completed questionnaires regarding their pain, stress, and satisfaction with the procedure.

#### 3) Control group

Participants in the control group underwent venipuncture without receiving warm therapy or digital distraction. The TEET device was used only as a conventional tourniquet to facilitate vein visualization. No thermal stimulation or distraction was applied. The same nurse performed IV catheterization using an 18-gauge angiocatheter. After catheter insertion, participants completed the same pain, stress, and satisfaction questionnaires used in the other groups.

### 4. Instruments

#### 1) Pain and stress

Pain and stress were each measured using a numeric rating scale (NRS). Participants were shown a 10 cm horizontal line, with 0 (no pain/no stress whatsoever) on the left end and 10 (very severe pain/very severe stress) on the right end. They were asked to indicate their current level of pain and stress experienced during IV catheterization. In addition, to assess pain tolerance, participants were asked to recall their most recent IV catheterization experience within the past six months and rate the pain and stress they felt at that time using the same NRS scale. These retrospective ratings were used as indicators of individual pain and stress tolerance levels.

#### 2) Participant satisfaction

After the intervention was completed, we used an instrument developed by Hur [16] and revised and improved by Lee [8] to measure participants satisfaction. This instrument consists of nine items in total, covering pain relief, overall satisfaction, and intention to reuse the device. Each item was rated on a 5-point Likert scale (from 1, “not at all,” to 5, “very much”), with higher scores indicating higher satisfaction. In terms of reliability, Cronbach’s α was .87 in a previous study [16] and .93 in our study.

#### 3) Practitioner satisfaction

To allow the practitioner to rate their satisfaction with the intervention after completion, we used an instrument developed by Hur [16] and revised and improved by Lee [8], consisting of nine items covering help with venipuncture, overall satisfaction, and intention to reuse the device. Each item was rated on a 5-point Likert scale (from 1, “not at all,” to 5, “very much”), with higher scores indicating higher satisfaction. In terms of reliability, Cronbach’s α was .87 in a previous study [16] and .90 in our study.

#### 4) TEET

TEET is a medical device that uses a TEE to facilitate vein access and alleviate pain during IV catheterization [16]. In conventional venipuncture procedures, a tourniquet is essential for dilating the vein [8]; TEET enhances this process by incorporating a TEE that promotes increased blood flow and further facilitates venous access. The TEE generates an electromotive force (EMF) through temperature differences and induces temperature changes when an external EMF is applied [8]. The device was specifically developed to provide stable warming at the venipuncture site.

**Figure 3.**
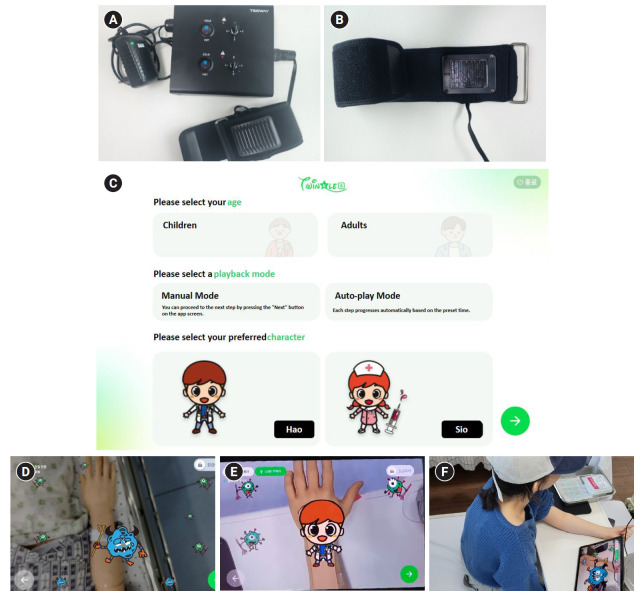
Overview of the TEET device and TWINKLE app implementation. (A) TEET device and its temperature controller; (B) TEET; (C) Main screen of the TWINKLE app; (D) TWINKLE app in use - Interface example 1; (E) TWINKLE app in use - Interface example 2; (F) Application of the TWINKLE app during venipuncture. TEET; Thermoelectric-element tourniquet; app = Application.

The development of the TEET involved collaboration among a nursing professor, a doctor of materials engineering, and an expert in flexible TEE. Together, they discussed the optimal design and made improvements based on existing South Korean patents (Nos. 10-1989908, 10-1829709, and 10-1689308). The TEET includes two flexible thermoelectric plates (5.5 cm × 3.5 cm), is designed to be applied approximately 10~12 cm proximal to the venipuncture site, and provides continuous, stable warming prior to IV catheter insertion (Figure 3-A). In this way, the TEET goes beyond the role of a simple physical tourniquet and, by using thermal stimulation to induce vasodilation, serves as an effective non-pharmacological intervention for relieving pain during venipuncture (Figure 3-B).

#### 5) TWINKLE app

TWINKLE is a digital distraction app that uses augmented reality. It was developed in 2024 through collaboration between the research team and a healthcare-based intervention company, Stress Solution (South Korea), specifically for use in clinical research. The development team included four PhD-level nursing professors, one music professor, and two application developers. The app is currently in the pilot testing stage and is not yet publicly available. It is used exclusively for research purposes in a controlled environment by trained healthcare professionals.

TWINKLE was designed to alleviate psychological anxiety and pain in patients undergoing IV catheterization. Augmented reality technology superimposes virtual elements onto live images of the real environment to create a more immersive experience [20]. This can reduce a patient’s fear of the catheterization process, enabling a more positive treatment experience than that of conventional methods. TWINKLE provides a visual guide for IV catheterization, encouraging the patient to focus on the digital content rather than the venipuncture site, thereby helping to alleviate their fear. The name TWINKLE comes from the sparkling star effect that appears after IV catheterization is successfully completed, which is intended to provide the patient with a positive emotional experience.

TWINKLE was developed in Unity, using camera image red, green and blue (RGB) analysis technology to play preloaded animations when specific colors are detected. The app uses keyboard input via a Bluetooth pedal so that healthcare workers can change screens while keeping their hands sterile. The user can replay or stop animations using the pedal, and an automatic replay function is also provided to maximize convenience for healthcare workers. Characters called Hao and Sio appear in the app to provide guidance and explanations of the IV catheterization process (Figure 3-C). Through the augmented reality experience (via a tablet PC screen connected to a camera), patients can view images of bacteria, and hear explanations about the catheterization procedure from animated characters (Figure 3-D). These features aim to provide distraction, alleviate tension, and increase comfort during catheterization. TWINKLE goes beyond simple visual distraction, providing an innovative intervention method using digital therapeutics in the process of IV catheterization. The design simultaneously considers the experiences of both the patient and healthcare worker, offering a more efficient, stable environment for IV catheterization (Figures 3-E, 3-F).

### 5. Data collection

Data were collected between 12^th^ December 2024 and 9^th^ January 2025. Prior to data collection, recruitment notices were posted on bulletin boards at Gimcheon University in Gimcheon, South Korea. Healthy adults who voluntarily contacted the research team after seeing the notice were screened according to inclusion and exclusion criteria.

All eligible participants received a face-to-face explanation of the study’s purpose, procedures, and ethical considerations from a trained research assistant in a controlled laboratory setting within the university. Written informed consent was obtained after the explanation.

For random allocation, we used the random number generator in Microsoft Excel 2019 and assigned participants to one of the three groups (experimental, comparison, or control) in order of arrival. Prior to data collection, all research assistants (registered nurses with at least three years of clinical experience) were trained in the study protocol to ensure intervention consistency. To maintain double-blinding, the assistants were not informed of the group assignments or study hypotheses.

### 6. Data analysis

The collected data were analyzed using SPSS version 30.0 (IBM Corp., Armonk, NY, USA). The participants’ general characteristics were analyzed using frequencies, percentages, means, and standard deviations. Homogeneity was tested using the χ^2^ test and analysis of variance (ANOVA). We used ANOVA to test the homogeneity of the dependent variables between the experimental, comparison, and control groups before the intervention. To test differences in pain during venipuncture, stress, and satisfaction after the intervention we first performed an ANOVA, and then analyzed significant results using the Bonferroni post-hoc test.

### 7. Ethical considerations

Before starting data collection, we received approval from the Institutional Review Board of Gimcheon University, located in Gyeongsangbuk-do, South Korea (GU-202407-HRa-06-P). To ensure the transparency and reliability of this clinical trial, we registered the study on the Clinical Research Information Service (KCT0010149). Participants were recruited offline and only those who voluntarily agreed to participate were included in the study. After receiving a thorough explanation of the study objectives and procedures, participants provided informed consent via a questionnaire. The questionnaire included detailed information about the study’s purpose, the protection of participants’ anonymity and personal information, and their right to refuse or withdraw from the study at any time. It was also clearly stated that they could stop the intervention during the study if desired. Additionally, we informed participants that all collected data would be used solely for research purposes, responses would be anonymized, and personal information would be stored in an encrypted file on a password-protected computer for three years before being permanently destroyed. A gift voucher was provided as a small token of appreciation upon completion of the study.

## RESULTS

### 1. Homogeneity of participants’ general characteristics and dependent variables before the intervention

There were no dropouts; all 93 participants completed the study and were included in the analysis. Before the intervention, there were no significant differences in gender, age, religion, or pain tolerance among the experimental, comparison, and control groups, indicating that the groups were homogeneous at baseline. In addition, participants’ pain and stress levels during their most recent venipuncture experience (within the past six months) were measured using a NRS to assess baseline sensitivity. There were no significant differences between the three groups in these prior venipuncture responses, confirming intergroup homogeneity in terms of both physical and psychological tolerance related to needle procedures (Table 1).

### 2. Patients’ pain, stress, and satisfaction and practitioners’ satisfaction post-intervention

#### 1) Pain

After the intervention, pain scores showed statistically significant differences between the experimental, comparison, and control groups (F = 5.68, *p* = .005). Post-hoc analysis revealed that the experimental group (2.48 ± 1.44) showed significantly lower pain scores than the control group (3.81 ± 1.58; *p* = .003; Table 2). Although the effect size was small (Cohen’s f = 0.11) [21], the absolute reduction of 1.33 points in pain scores between the experimental and control groups may still be considered clinically meaningful.

#### 2) Stress

Similarly, post-intervention stress scores showed statistically significant differences between the experimental, comparison, and control groups (F = 9.42, *p* < .001). Post-hoc analysis revealed significantly lower stress scores in the experimental group (2.10 ± 1.22) compared to the control group (3.74 ± 1.57; *p* < .001; Table 2). Cohen’s f = 0.17 indicated a small-to-moderate effect size [21], suggesting that the intervention had a meaningful psychological benefit in reducing stress.

#### 3) Participant satisfaction

After the intervention, participant satisfaction showed statistically significant differences between the experimental, comparison, and control groups (F = 17.65, *p* < .001). In post-hoc analysis, the experimental group (40.32 ± 5.41) showed significantly higher satisfaction than the comparison group (35.77 ± 5.36; *p* = .012) and the control group (31.16 ± 7.25; *p* < .001). There were also significant differences between the comparison and control groups (*p* = .011; Table 2). With a Cohen’s f = 0.28, the effect size was moderate [21], reflecting a meaningful improvement in perceived participant satisfaction due to the combined intervention.

#### 4) Practitioner satisfaction

After the intervention, practitioner satisfaction showed statistically significant differences between the experimental, comparison, and control groups (F = 67.91, *p* < .001). In post-hoc analysis, the comparison group (42.74 ± 3.39) showed significantly higher satisfaction than the experimental group (37.03 ± 4.21; *p* < .001) and the control group (31.39 ± 3.87; *p* < .001). There were also significant differences between the experimental and control groups (*p* < .001; Table 2). Cohen’s f = 0.60 indicated a large effect size [21], suggesting a strong difference in nurse satisfaction, especially when warm therapy alone was applied.

## DISCUSSION

This study investigated the effects of an intervention combining warm therapy (using a TEET) and digital distraction (using the TWINKLE app) during IV catheterization of healthy adults on patient pain, stress, and satisfaction and practitioner satisfaction, and compared these effects with those of existing intervention methods. We found that the experimental group (using both the TEET and TWINKLE app), showed significantly lower pain and stress scores, as well as the highest patient satisfaction scores.

The pain scores of the experimental group were significantly lower than those of the control group, and the pain alleviating effect was stronger than that of the comparison group. After the intervention, the results demonstrated the effectiveness of the intervention for pain relief during venipuncture. These findings are consistent with previous studies reporting the effectiveness of physical and distraction-based interventions for pain relief. Raafat et al. [22] evaluated the effect of warm application on propofol injection pain in surgical patients by applying a warm compress (rubber gloves containing 200 mL of 40°C water) to the injection site for five minutes before the procedure. They concluded that warm application produced a pain-reducing effect comparable to lidocaine, suggesting the efficacy of heat therapy in invasive procedures. While their study focused on surgical patients receiving anesthetics, the present study extends the applicability of warm therapy to a more general population by evaluating its effects during routine IV catheterization in healthy adults.

Similarly, Piskorz and Czub [14] reported that pediatric patients undergoing venipuncture experienced significantly reduced pain and stress when distracted using a head-mounted VR game based on multiple object tracking. In contrast, the present study applied a tablet-based augmented reality app (TWINKLE) as a distraction tool during IV catheterization in adults, allowing for easier implementation in clinical practice. Furthermore, unlike the previous studies that evaluated each intervention in isolation, our study combined digital distraction and warm therapy, providing evidence for a synergistic effect that simultaneously addresses both physical and psychological components of procedural pain.

Warm therapy’s capacity to relax the skin and vasculature and increase the blood flow effectively reduces pressure and resistance during needle insertion [22]. It also acts by inhibiting neurotransmission of pain and decreasing the sensitivity of nerve receptors [23]. The additional use of the distraction app TWINKLE appeared to contribute to pain relief in the present study. This adds support to a meta-analysis of the effects of distraction therapy on pain during IV catheterization among children, in which distraction interventions such as VR, music, video games, and blowing bubbles were reported to effectively reduce pain during venipuncture [12]. These results suggest that combining TEET-based warm therapy with the TWINKLE app may enhance pain relief by simultaneously promoting physical comfort and psychological relaxation, offering a more effective strategy than using either intervention alone. While the physical stimulus of TEET inhibits neurotransmission of pain, the TWINKLE app redirects participants’ attention, providing a complementary effect in regulating psychological pain perception. Hence, non-medicinal pain interventions could be even more effective when physical and psychological responses are targeted simultaneously.

Regarding stress, our intervention effectively reduced psychological tension, with mean post-intervention stress scores indicating a significant decrease in stress levels in the experimental group. These results demonstrate that combining TEET warm therapy with the TWINKLE app effectively alleviates psychological stress in patients undergoing venipuncture. With very few previous studies validating the effects of warm therapy on stress reduction during IV catheterization, our results mirror those found in Lee et al.'s study [24] on myalgia patients, which reported that warm therapy effectively stabilized the autonomic nervous system and relieved tension. Thus, warm stimuli appear to serve both local physical effects and broader psychological benefits, reinforcing the notion that the combined intervention of TEET and TWINKLE could alleviate both tension and stress during venipuncture. TWINKLE is likely to have played an important role in reducing participants’ anxiety and regulating their stress response. This combined approach overcomes limitations associated with conventional physical interventions, which may struggle to alleviate psychological anxiety effectively.

Participant satisfaction scores were notably high in the experimental group, indicating that the combined intervention enhanced the overall participant experience. It is likely that satisfaction was increased due to the simultaneous implementation of pleasant physical stimuli (using the TEET) and psychological distraction (from the TWINKLE app). Previous studies investigating the effects of warm therapy using TEET during venipuncture reported similar findings, noting significantly higher satisfaction in the TEET group compared to the control [8]. Our results reinforce this, suggesting that the combination of physical pain relief from TEET and the psychological relaxation effects of the TWINKLE app contributed meaningfully to improving participant experiences during IV catheterization. This study is important as it directly measured participant satisfaction during venipuncture interventions; such evaluations are limited in previous research.

Notably, among practitioners, satisfaction scores were highest in the comparison group utilizing only warm therapy, followed by the experimental and control groups. This finding suggests that nurses found venous access easier when only warm therapy was applied, aligning with earlier studies that reported higher practitioner satisfaction with warm therapy [8]. On the other hand, the experimental group demonstrated significantly lower stress and higher participant satisfaction compared to both the comparison and control groups. These findings suggest that while TEET alone is effective in enhancing physical comfort and procedural efficiency, the addition of TWINKLE further amplifies psychological benefits such as anxiety reduction and emotional satisfaction. This highlights the complementary roles of the two interventions: TEET primarily addresses physiological discomfort, whereas TWINKLE contributes to psychological relief, making the combined approach more integrated in its nursing effect. However, practitioner satisfaction was slightly lower in the experimental group, which additionally received distraction therapy using the TWINKLE app. We suggest that the additional work burden for nurses of having to implement the digital intervention could have decreased their satisfaction relative to the comparison group. Therefore, future studies should aim to streamline the use of the TWINKLE app in clinical settings, reducing the associated workload for nurses and enabling them to focus more effectively on direct patient care. Our findings indicate that rather than uniformly applying the TWINKLE app to all patients, personalized approaches should be adopted for patients demonstrating high psychological anxiety during venipuncture. Screening for high-stress patients may enhance the effectiveness of nursing interventions, particularly in creating patient-centric protocols while minimizing the burden on nursing staff.

In summary, this study is valuable because it is one of the first to apply warm therapy with TEET and distraction therapy using the TWINKLE app to adults, and to analyze the effects on pain and stress relief and improving satisfaction. In particular, our work is distinct from previous studies because we combined a digital distraction technique with physical warm therapy. Healthy adults were selected in this study to first assess responses to the intervention in a general population, minimizing confounding factors. Based on these findings, future research is planned to apply the combined intervention to various clinical populations.

Nonetheless, this study has several limitations that should be acknowledged. First and foremost, participants were all healthy adults, which may limit the generalizability of our findings to broader clinical populations. The unique responses of individuals with chronic illnesses or varying health conditions should be investigated in future studies to assess the applicability of this intervention in diverse patient profiles. Additionally, the sample was predominantly female, highlighting the need for future research to include a more gender-balanced population to evaluate potential gender differences in response to these interventions. This acknowledgment of diversity is essential for developing inclusive treatment protocols that can effectively address the needs of all patients. Future research should also focus on evaluating the long-term effects of combined interventions using TWINKLE and TEET in varied clinical settings, as well as exploring different patient demographics and their unique responses to these strategies. Moreover, understanding the implications of these interventions on nurses’ work efficiency and overall job satisfaction will further solidify their integration into everyday clinical practice. Strategies to maximize usability in clinical settings need to be explored to enhance the effective implementation of digital interventions, ensuring they add value to patient care while minimizing the workload for nursing staff.

This study has several strengths. It is one of the first trials to integrate thermoelectric warm therapy with augmented reality-based distraction for venipuncture in healthy adults, providing empirical evidence for the synergistic benefits of physical and psychological interventions. However, it also has limitations. The sample consisted solely of healthy adults, limiting the generalizability of the findings to broader patient populations. Additionally, the intervention was applied in a controlled environment by trained practitioners, which may differ from real-world clinical settings. Future studies should consider including clinical populations and evaluating the intervention in diverse healthcare environments.

## CONCLUSION

This study verified the effectiveness of a combined intervention using TEET and the augmented reality-based TWINKLE app in reducing pain and stress, and improving participant satisfaction during IV catheterization. The experimental group, which received both warm therapy and digital distraction, showed significantly better outcomes than the comparison or control groups, suggesting a synergistic effect when physical and psychological interventions are applied together.

While warm therapy alone enhanced practitioner satisfaction by improving the ease of venipuncture, the addition of distraction increased the complexity of the procedure. This highlights the need for individualized application based on participant anxiety levels and workflow adjustments to support clinical feasibility.

Overall, this study contributes to evidence-based nursing by presenting a multidimensional, non-pharmacological strategy to enhance the participant experience during venipuncture. Given that the TWINKLE app is currently at the prototype stage and restricted to research settings, further development is needed to enhance its clinical usability and cost-effectiveness. Future research will focus on refining the user interface and reducing operational complexity to better integrate it into routine clinical workflows. Additionally, TWINKLE is expected to be adapted for use in specific patient groups such as pediatric inpatients and preoperative patients undergoing IV procedures, across diverse clinical settings and populations. Further studies should focus on validating the effectiveness of the combined intervention in diverse clinical populations and real-world settings, while refining implementation strategies to enhance usability in routine practice.

## Figures and Tables

**Table 1.** Homogeneity Test for General Characteristics and Dependent Variables between Groups (N = 93)

Variables	Categories	Experimental group^†^ (n = 31)	Comparison group^‡^ (n = 31)	Control group^§^ (n = 31)	χ ² or F	*p*
Sex	Men	8 (8.6)	10 (10.8)	5 (5.4)	1.09	.341
	Women	23 (24.7)	21 (22.6)	26 (27.9)		
Age (yr)		23.03 ± 2.85	22.23 ± 1.48	21.94 ± 1.03	2.63	.077
Religion	Yes	8 (8.6)	8 (8.6)	7 (7.5)	0.06	.946
	No	23 (24.7)	23 (24.7)	24 (25.9)		
Pain tolerance	Strong	9 (9.7)	12 (12.9)	12 (12.9)	1.64	.200
	Moderate	12 (12.9)	15 (16.1)	12 (12.9)		
	Weak	6 (6.5)	3 (3.1)	6 (6.5)		
	Very weak	4 (4.3)	1 (1.1)	1 (1.1)		
Previous venipuncture pain (NRS)		4.87 ± 2.50	4.23 ± 2.11	4.45 ± 2.14	0.65	.523
Previous venipuncture stress (NRS)		4.23 ± 2.06	3.84 ± 1.92	3.48 ± 1.98	1.08	.344

Values are presented as the mean ± standard deviation or n (%). NRS = Numerical rating scale.

^†^Experimental group = TWINKLE app with warm therapy; ^‡^Comparison group = Warm therapy only; ^§^Control group = No intervention.

**Table 2.** Post-test Comparison of Pain, Stress, Participant Satisfaction, and Nurse Satisfaction among Three Groups (N = 93)

Variables	Experimental group^†^	Comparison group^‡^	Control group^ §^	F	*p*	Effect size (Cohen’s f)	Post-hoc test (Bonferroni test)
Pain (NRS)	2.48 ± 1.44	3.10 ± 1.62	3.81 ± 1.58	5.68	.005	0.11	† < §
Stress (NRS)	2.10 ± 1.22	2.94 ± 1.65	3.74 ± 1.57	9.42	< .001	0.17	† < §
Participant satisfaction	40.32 ± 5.41	35.77 ± 5.36	31.16 ± 7.25	17.65	< .001	0.28	† > ‡ > §
Nurse satisfaction	37.03 ± 4.21	42.74 ± 3.39	31.39 ± 3.87	67.91	< .001	0.60	‡ > † > §

Values are presented as the mean ± standard deviation. NRS = Numerical rating scale.

^†^Experimental group = TWINKLE app with warm therapy; ^‡^Comparison group = Warm therapy only; ^§^Control group = No intervention.

## Data Availability

The data that support the findings of this study are available from the corresponding author upon reasonable request.
